# Sanitary Condition of Western Steamers

**Published:** 1855-03

**Authors:** 


### EDITORIAL DEPARTMENT.

### SANITARY CONDITION OF WESTERN STEAMERS.

This subject has not received that attention which its importance demands. The very frequent occurrence of disease on board these water craft, entitle them in our opinion to the unenviable distinction of floating "*pest-houses.*" We unhesitatingly declare it to be our conviction, that there was scarcely a boat from the south which landed at our port during the last season, from early spring until late in the fall, on board which there was not one or more sick persons, and oftimes a corpse requiring the rites of sepulture. For several of the previous years, the frequent occurrence of sickness in some form or other upon our steamers, became so generally known, that it was the subject of universal remark, and with many of severe animadversion. A serious dread was awakened in the public mind in adopting this mode of travel, and when the extended system of railroads had well nigh covered in a net-work the states of Ohio and Indiana, and promised soon to throw its meshes over us, congratulations were indulged in, that soon we would have a safe, comfortable and expeditious mode of reaching the East without the hazard of collapses, snags or fire.

Since then, and particularly during the past year, our experience and observation are not in favor of either the comfort or safety of railroad travel, although more rapid in movement. On account of the numerous accidents upon rail cars, and the startling accounts of "mangling" and "maiming," blackening the columns of the public journals with their horrid recitals, the public attention is again turned to steamers, as decidedly the safer of the two modes of travel, though not quite so expeditious. Certain it is that there has been a less number of accidents during the past year than usual upon our western waters, while upon railroads they have fearfully multiplied.

The reason for this exemption may be found to be two-fold: *First*, in the rigid exaction of the law requiring the highest capacity and the most perfect sobriety of the engineers: and, *Second*, the fact that the commerce upon our rivers was limited in some, and entirely suspended in others. The last mentioned circumstance doubtless increased the travel upon the railroads, for the vast emigration to the west from the east, sought these thoroughfares rather than endure a long, laborious and protracted journey over-land, liable to disease because of the exposures they must endure. Now that the journey can be performed, from the extreme east to the banks of the Mississippi in three days, whereas by wagons it would require almost as many months, the achievement is a great one, and the results incalculable. With this increase of numbers upon the railways, there would be an increased liability to accident arising out of derangements in their time of running; and the greatly increased demand for vehicles and propelling power would reconcile the use of those means of conveyance which a prudent regard for the lives of our fellow beings, should have suggested the propriety of being withheld. That these conscientious scruples, if they were ever felt, were in many instances disregarded during the last season there is not a doubt.

In view then of the fact that the public attention is again turned in the direction indicated above, and in view also of the fact that on the part of those engaged in the management of all the various thoroughfares, there is a stolid indifference and a criminal contempt evinced for the safety of the wayfarer, who has put his life in their hands for safe keeping, we say in view of these things, it is the duty of that public to investigate and as far as possible to correct these evils, diminish the number on the category, and remove out of the way those wholly needless and avoidable. And we look upon the public journals, particularly those devoted to the discussion of the best means of preserving health and life, to be solemnly called upon to point out, and expose certain criminal omissions of duty which it would for the benefit of all to observe.

Without undertaking to indicate the mode by which certain existing evils may be removed, it is sufficient for our purpose to point out those evils in order that some mode of reformation may be adopted by those who may be authorized to execute it. The moral delinquencies of officers and owners, resulting often in collisions, explosions, burnings and snaggings, are subjects which belong to others to discuss, and evils which require their own and appropriate remedies. The construction and management of these craft with reference to comfort and health will demand our attention now.

The term "western waters," is understood to mean the Mississippi river, beginning at its mouth and extending to the head of the steamboat navigation of each of its numerous tributaries. Some of these constitute channels of conveyence from the east to the fertile valleys of the west, while others penetrate into the very heart and centre of these productive and fertile regions, to which the tide of emigration is yearly flowing in living streams.

Not only are the eastern states contributing to this, but foreign countries are furnishing largely of material to swell the human current. The immigrants from abroad, no matter at what sea-port they land, are found on the way to their destination, upon the main trunk of the Mississippi or some of its tributaries. The situation in which they land, or a large majority of them, their condition while traveling, and their mode of living, under *any* circumstances, are beyond all description wretched and filthy. When on their "winding way," however, to their new homes upon these steamers, they constitute, as it were, so much inflammable matter, which is easily ignited by the warmth of a pestilential breath.

To this tide of immigration, we must add the travel, which largely swells the numbers of this vast moving mass of human beings. These all meet upon the same boats, those who respect health and propriety so far as to avoid filthy habits, and those of the above mentioned class. These do not mingle often, but they breathe the same atmosphere, which is often rendered still more impure, if possible, by those who contemn personal cleanliness, comfort and health. In addition to this, there is a miscellaneous collection of freight. Not only do boats take freight and passengers ; but they also take domestic animals of all kinds. The boat itself, is composed of three apartments, ranged one above another—the lowest of which is the "hold," except a small space between the floor of the hold, and the true bottom of the boat of from four to six inches. Above the hold, is the deck, which is sub-divided again into passenger deck and the boiler deck, between which are the engines. Above these again, the "cabin." Surmounting these is still another, which, because it is a kind of territorial extension, is dignified with the title of "Texas."

Merchandise, grain of all kinds, and everything usually carried upon boats, are stowed in these holds. Leakage from molasses, spirits, oils, &c., runs upon the floor of these holds and percolates through the seams into the space below. These materials mingle with the "*bilge water*," which constitutes a heterogeneous mass of a most foul and noxious character. The emanations from this rise upward and diffuse themselves through all parts of the deck. This foul mixture extends from *bow* to *stern*; and we have been assured, by persons of abundant opportunity for knowing, that this is often so offensive, as to make it unpleasant and hazardous to remain in these "holds," for any considerable length of time. These foul gases are not confined to the decks alone, but they diffuse themselves more or less over the entire boat.

The "passenger deck" is an area of from sixteen to twenty feet square, and in the centre is a *huge engine*, often well broken up, called a cooking stove. This is saturated with oils, and when heated, sends forth vapors unpleasant and suffocating. On each side there is a kind of rack, black with smoke, and soaked with grease, intended as a kind of frame for beds or "bunks." This is divided into apartments, and ranged one above another. These frames are often further and still more *richly* endowed with certain elements of comfort in the shape of a filthy mattrass, and perhaps a blanket or quilt, in even a worse condition. Immediately in the rear of the boat is a wide aperture which is really the only element of comfort connected with this part of the deck, and even this becomes a source of discomfort and annoyance in stern wheel crafts, because of the mist blown in by the action of the wheels. The space constituting the deck proper, is often encroached upon and materially limited in size by fortifications composed of boxes of goods, crates filled with straw, hogsheads filled with ware and damp straw, grain sacks, beds and bedding of emigrants; these piled up to the floor of the cabin, constitute quite a formidable obstruction. A narrow passage is left by which to reach the interior of this fortified area which, when reached, is found filled with human beings of all ages, sexes, and usually in the very worst possible condition. The air is surcharged with noxious vapors, and is in many in stances intolerably offensive. Different kinds of food are in process of preparation upon a highly heated stove, necessary even in midsummer, and the air of this limited and crowded apartment is filled with a compound aroma made up of food in process of cooking, odors of carbonizing oils, the foul emanations from the fecal evacuations of children, the stench from the excrement of cattle or horses which often flank decks on each side, the exhalations from decaying straw and decomposing grain, some of which has been littered and strewed over a humid deck floor, constitute no exaggarated picture of the condition of very many of the steamers which have arrived at our port during the past year.

The reader will conclude, that in the above, there are found abundant causes for the much sickness ocurring upon these boats without referring to the additional fact, the heat of the boilers and the humidity arising from constantly escaping steam, which are driven back by the current occasioned by the onward movement of the boat, and which under the circumstances might have remained latent.

And then again, coming up from the hold below through every cranny of the deck floor, are the foul emanations engendered there to join the cohorts of death dealing emissaries which have already been marshalled to the attack.

The water of the Mississippi and its tributaries, particularly from the north, constitutes a large share in the production of disease. An analysis shows the existence of lime in several forms, vegetable extraction and animal matters in solution, of the latter even some are suspended and not dissolved.

These are highly irritating to the alimentary canal of persons whose digestive organs are impaired, and in a particular manner in children. In these last, gastric and enteric disturbances are very readily produced. Independent of the above detailed influences, gastro-enteric difficulties arè often produced by an indulgence in its use, particularly by those unused to it.

The habits of the emigrant when on board these steamers, not only predispose to, but excite disease. This embraces considerations of cleanliness and diet. The exhilerating influences arising from the rapid movement of the vessel, and the quick and successive changes of scene are circumstances well calculated at first, to impart an appetite which is usually freely indulged and gratified. If the journey be some days protracted, the digestive organs are clogged and impaired in their function which, added to the causes heretofore mentioned, largely augment the tendency to disease and *per consequence* contribute to the number of cases.

Again, the diet may be deficient in *quality* as well as in *quantity*. The circumstances in which they are placed forbid the possibility of a good preparation. If it were even proper in quality, quantity and mode of preparation, the digestive powers fail, as already observed, and denied the opportunity of exercise in pure air they would be found in the best possible condition for the production of disease from causes intrinsic or extrinsic. for it is a fact well known, that the indulgence in food of bad quality or defective in quantity is more seriously felt than elsewhere on board ship, in prisons, in asylums, or any other close habitation in which a large number of human beings are crowded, and where the due amount of air and exercise cannot be indulged in.

The cabins, though better, are yet not free, in many instances from gross outrages upon certain sanitary rules and requirements. The state rooms are too small, ventilated apparently upon the principle that fresh air was too valuable, cost too much, and that it could not be afforded. The beds are too often filthy, and yet, although these rooms are too small for the health and comfort of one individual, two are crowded in, not to sleep in bunks of equal height on terms of *perfect equality*, but one is placed above the other, the individual flattered by promotion, to receive in addition the benefits and favors of the foul expired air of the *humble* and *lowly* just under him.

We could dwell at great length upon this subject; but we will dismiss it in the hope that enough has been said to call the attention of the profession to the importance and necessity of a change. It is incumbent particularly upon the profession of the west, because the numerous travelers and immigrants bound for the western states who have either died upon these boats, or immediately after landing, are referred to as instances of the great unhealthiness of the climate, when if our medical men would but follow our example, penetrate into the lower apartments of these boats, search out and expose every criminal neglect of human comfort and these irrefragable evidences of indifference to human life, such an agitation would lead to greater security by the establishment of the most rigid sanatory laws and regulations.

